# Tunable Dipole Surface Plasmon Resonances of Silver Nanoparticles by Cladding Dielectric Layers

**DOI:** 10.1038/srep12555

**Published:** 2015-07-28

**Authors:** Xiaotong Liu, Dabing Li, Xiaojuan Sun, Zhiming Li, Hang Song, Hong Jiang, Yiren Chen

**Affiliations:** 1 State Key Laboratory of Luminescence and Applications, Changchun Institute of Optics, Fine Mechanics and Physics, Chinese Academy of Sciences, Changchun 130033, People’s Republic of China; 2 University of Chinese Academy of Sciences, Beijing 100039, People’s Republic of China

## Abstract

The tunability of surface plasmon resonance can enable the highest degree of localised surface plasmon enhancement to be achieved, based on the emitting or absorbing wavelength. In this article, tunable dipole surface plasmon resonances of Ag nanoparticles (NPs) are realized by modification of the SiO_2_ dielectric layer thicknesses. SiO_2_ layers both beneath and over the Ag NPs affected the resonance wavelengths of local surface plasmons (LSPs). By adjusting the SiO_2_ thickness beneath the Ag NPs from 5 nm to 20 nm, the dipole surface plasmon resonances shifted from 470 nm to 410 nm. Meanwhile, after sandwiching the Ag NPs by growing SiO_2_ before NPs fabrication and then overcoating the NPs with various SiO_2_ thicknesses from 5 nm to 20 nm, the dipole surface plasmon resonances changed from 450 nm to 490 nm. The SiO_2_ cladding dielectric layer can tune the Ag NP surface charge, leading to a change in the effective permittivity of the surrounding medium, and thus to a blueshift or redshift of the resonance wavelength. Also, the quadrupole plasmon resonances were suppressed by the SiO_2_ cladding layer because the dielectric SiO_2_ can suppress level splitting of surface plasmon resonances caused by the Ag NP coupling effect.

Surface plasmons are attracting increasing interest because of their localised surface plasmon resonance (LSPR) properties, which induce large local electromagnetic field enhancements and can thus be used to improve the performance of optoelectronic devices^1^,^2^,^3^,^4^,^5^. When the energy of the incident or emitted light coincides with the localised surface plasmon energy, an effective energy transfer occurs from the light beam to the local surface plasmons (LSPs). Once transferred, this energy excites the LSPs, which can effectively enhance the absorbing or emitting photons, leading to the strongest possible coupling. Therefore, the degree of peak energy matching between the resonance energy of the LSPs and the energy of the light determines the localised surface plasmon enhancement factor. The closer that these two energies are, the more that the resulting performances of the optoelectronic devices will be enhanced.

The resonant wavelengths of LSPs can be tuned using the shape, size, and type of metal nanoparticles (NPs)^6^,^7^,^8^. Modification of the medium has also been considered to be a useful method for tuning of the resonance wavelength of LSPs because the LSPR wavelength maximum, λ_max_, is sensitive to the dielectric constant ε^9^,^10^,^11^. In nanoplasmonic enhancement applications, and especially those in the near violet and visible region, Ag nanoparticles are considered to be better than other metallic materials because they exhibit reduced parasitic absorption, which comprises unwanted losses that arise from resonant coupling of the incident sunlight to the nanoparticles^12^,^13^. It has previously been reported that this resonant scattering effect can be as much as 80% for Ag NPs with diameters as low as 50 nm^14^. To date, Ag has been used successfully in ZnO- and GaN-based light-emitting diodes (LEDs), solar cells, and detectors^15^,^16^,^17^. However, there is still considerable room to increase the efficiency of the devices by tuning the resonant wavelengths of the LSPs to match the absorbing or emitting wavelengths of the devices, especially in the case of GaN-based blue LEDs. However, most research in the area of surface plasmon resonance tunability is focused on achievement of a wide tunable λ_max_ range rather than the question of how to control the resonant wavelengths of LSPs easily and stably for GaN-based blue LEDs in the wavelength range from 400 nm to 500 nm.

In this article, the tunable dipole surface plasmon resonances of Ag NPs between 400 nm and 500 nm were investigated using sapphire as the substrates. The results showed that the dipole surface plasmon resonances of the LSPs can be shifted easily and stably between 410 nm and 490 nm by adjusting the thickness of the SiO_2_ beneath and overcoating the Ag NPs. The time-domain and frequency-domain finite-element methods were used to explain the tunable mechanism. The results presented here represent a further step towards greater efficiency enhancement for GaN-based LEDs.

## Results

**Figure 1:**
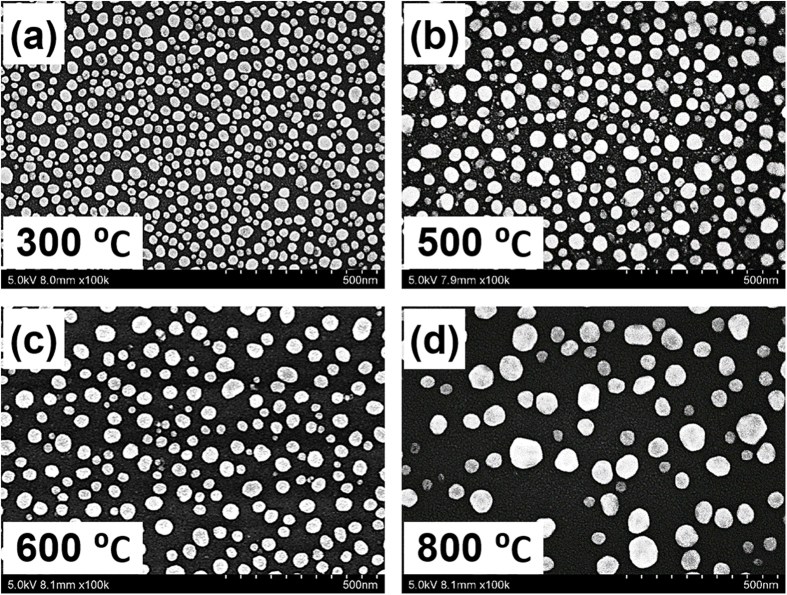
Morphologies of Ag NPs after thermal annealing treatments at different temperatures. (**a**) 300 °C, (**b**) 500 °C, (**c**) 600 °C, and (**d**) 800 °C.

Ag NPs with various sizes and distributions are fabricated by electron beam evaporation followed by a thermal annealing process. The morphologies of the Ag NPs after annealing at various temperatures are shown in Fig. 1. The thermal annealing treatment determines the Ag NP size and the spacing between adjacent Ag NPs. At an annealing temperature of 300 °C, the average size of the Ag NPs is approximately 60 nm, with the gap distance ranging from 10 nm to 60 nm. When the annealing temperature increases from 300 °C to 800 °C, the size of the Ag NPs also increases from 60 nm to 100 nm. At the same time, the density of the Ag NPs decreases and the Ag NPs then exist mainly in isolation or as dimers and trimers after annealing at 500 °C, 600 °C, and 800 °C. However, for the dimers or trimers, the gap distance between adjacent Ag NPs also ranges from 10 nm to 60 nm. Because the Ag NPs form by thermal annealing, the surface tension and the recrystallisation process make the size and distribution of the Ag NPs appear to be random.

**Figure 2:**
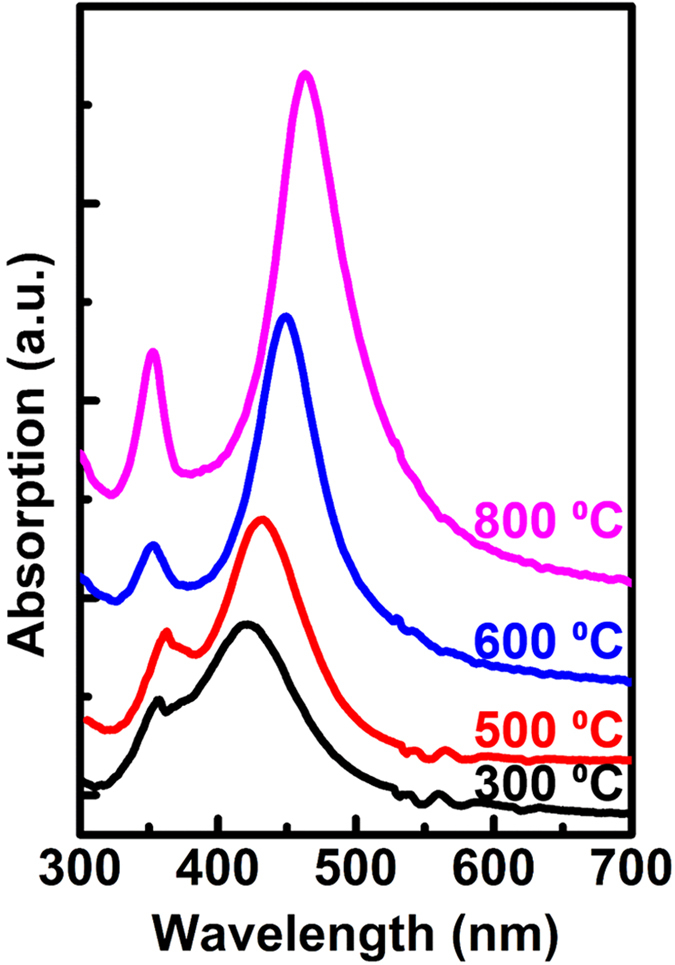
Extinction spectra of Ag nanoparticles for various thermal annealing treatment temperatures.

To determine the effects of the different sizes and distributions of Ag NPs on the resonant wavelengths of the LSPs, the extinction spectra were measured for all the samples, as shown in Fig. 2. The dipole resonant wavelength of the Ag NPs is seen to shift from 425 nm to 470 nm when the annealing temperature changes from 300 °C to 800 °C. This result suggests that the resonant wavelengths of LSPs can be tuned based on the size and distribution of the Ag NPs. Because the gap distance between adjacent Ag NPs ranges from 10 nm to 60 nm, the Ag NP size dominates the dipole resonant wavelength, and the larger the Ag NP is, the greater the redshift that occurs in the dipole resonant wavelength. Because the distance between charges on opposite surfaces of the Ag NP increases as the NP diameter increases, the restoring force is reduced, leading to a redshift in the resonant wavelength of the LSPs for larger NPs. In addition, Fig. 2 also shows that the quadrupole plasmon resonant wavelength exists at approximately 360 nm for all four samples, and the quadrupole plasmon resonance intensity is enhanced with increasing annealing temperature, which is mainly because the charge redistribution and the energy level splitting effect increase with increasing Ag NP size^18^. However, this enhancement of the quadrupole plasmon resonance is located outside the expected region of 400 nm to 500 nm. Therefore, the dipole resonant wavelength of the LSPs can be tuned by 45 nm in the blue region by modifying the size of the Ag NPs. However, the resonant wavelengths of the LSPs cannot be controlled precisely by this method because of random recrystallisation of the Ag NPs.

**Figure 3:**
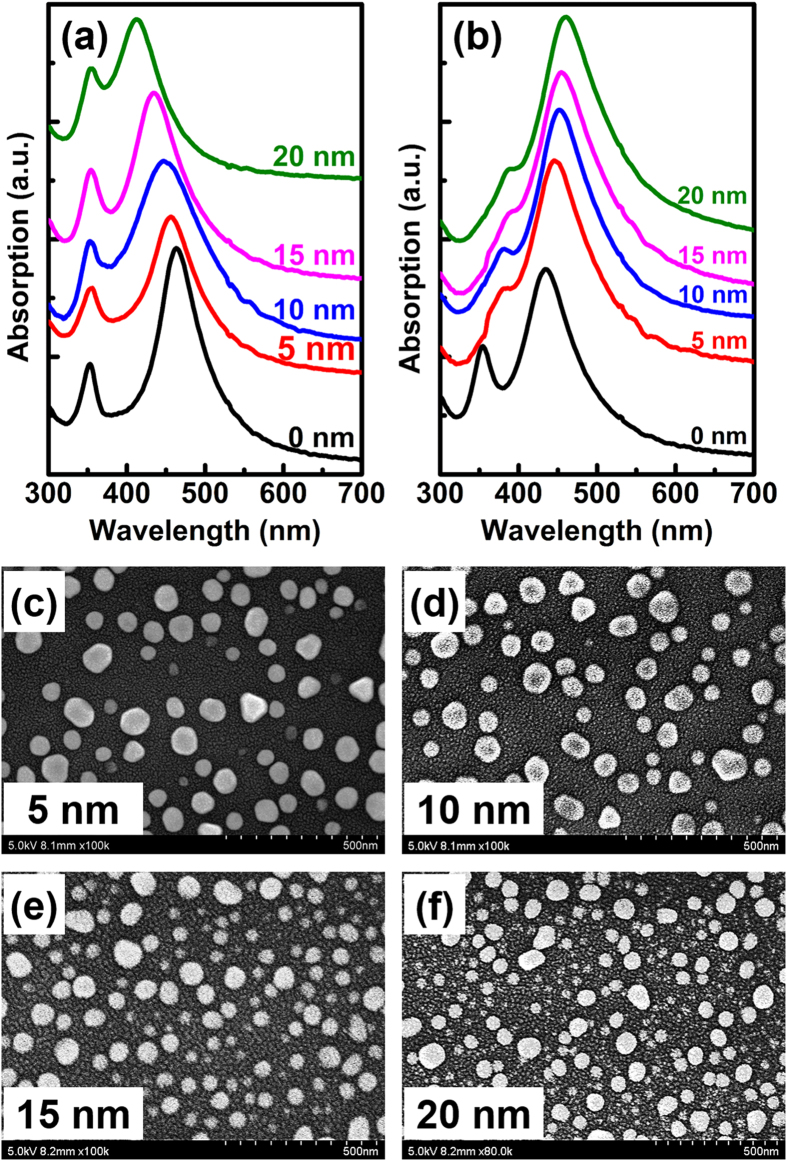
Fabrication of Ag-SiO_2_ nanostructures. Ag NPs are annealed at 800 °C for all the samples (**a**) Extinction spectra of Ag NPs with different SiO_2_ thicknesses beneath the nanoparticles, ranging from 0 nm to 20 nm. (**b**) Extinction spectra of Ag sandwich structures with 10 nm of SiO_2_ beneath the Ag NPs and various thicknesses of SiO_2_ overcoating the NPs, ranging from 5 nm to 20 nm. (**c–f**) Morphologies of sandwiched Ag nanoparticles cladding with different SiO_2_ thicknesses of (**c**) 5 nm, (**d**) 10 nm, (**e**) 15 nm, and (**f**) 20 nm.

For more accurate control of the resonant wavelengths of LSPs, SiO_2_ was grown beneath the Ag NPs with various layer thicknesses, and the extinction spectra of these samples were also studied. Figure 3(a) shows the extinction spectra measured when different SiO_2_ layer thicknesses are grown beneath the Ag NPs. All the Ag NPs in each samples were annealed at 800 °C. The dipole resonant wavelength of the Ag NPs shifts from 470 nm to 410 nm as the SiO_2_ thickness increases from 0 nm to 20 nm. The effects of SiO_2_ overcoating of Ag NPs on the dipole resonant wavelengths of the Ag NPs were also studied. Figure 3(b) shows typical extinction spectra of samples with sandwiched structures, which include 10 nm of SiO_2_ beneath the Ag NPs and various SiO_2_ thicknesses overcoating the NPs. The figure shows that the dipole resonant wavelength of the Ag NPs shifts from 450 nm to 490 nm when the SiO_2_ overcoating thickness changes from 0 nm to 20 nm. The excitation spectra of many individual samples are measured, which exhibit that although the Ag NPs in the individual samples are random and not exactly identical to each other, they has the same resonant wavelength under the same condition because given the same fabricating process, such as the same thickness and the annealing temperature, the Ag NPs have the same size and distribution in statistics. These results indicate that the dipole resonances of the Ag NPs can be tuned in a wide range between 410 nm to 490 nm by introducing SiO_2_ media with different thicknesses.

To determine the reasons for this change in the effective permittivity, the morphologies of the sandwiched Ag NPs that were clad with SiO_2_ were studied by scanning electron microscopy (SEM), as shown in Fig. 3(c)-Fig. 3(f), which are the evolution of Ag NPs cladding with SiO_2_ on the same sample by interrupting the depositing process of SiO_2_ by 4 times, each time of which is 5 nm. The results show that the SiO_2_ media under 20 nm were nanoparticles rather than a continuous film, and that the proportion of SiO_2_ occupying the surrounding medium rises with increasing SiO_2_ thickness, thus producing a higher contribution to the effective permittivity. Noted that as increasing the thickness of SiO_2_, there are some small particles appears at the interval the Ag NPs. They are the gathered SiO_2_ NPs being enlarged in visual because SiO_2_ is dielectric and the electron are more likely to accumulate around them during the SEM measurement.

In contrast, the intensity of the quadrupole resonance of Ag NPs decreases as the thickness of the SiO_2_ overcoating the Ag NPs increases, as shown in Fig. 3(b). The dielectric SiO_2_ cladding layer can suppress the asymmetric distribution of the surface charges and the cleavage of the surface plasmon resonance caused by interparticle coupling interaction of the Ag aggregates. Therefore, a thicker SiO_2_ cladding layer leads to reduced quadrupole resonance intensity for the Ag NPs.

In addition, we repeated our experimental measurement of these Ag NPs after one months, six month and even one year. The results show that, for the bare Ag NPs, compared with that of the first time measurement once they are fabricated, the dipole surface plasmon resonances wavelength of Ag NPs turned up to be redshift. The degree of the redshift increases as the time goes on, about 20 nm redshift for the bare Ag NPs after one year because of the oxidized of Ag NPs. However, for the samples of Ag NPs cladding by SiO_2_, the dipole surface plasmon resonances wavelength nearly keeps the same after an amount of time, even after one year, mainly because SiO_2_ protects the Ag NPs form being oxidized by the air. These results also prove that the method of tuning the dipole surface plasmon resonance wavelength of Ag NPs by cladding SiO_2_ is more stable and precise than that of changing the size, distribution of bare Ag NPs.

## Discussion

Several researchers have studied modification of the surrounding media to tune the resonance wavelengths of the Ag NPs, and the SiO_2_ dielectric layer has also been considered. In these studies, Maxwell-Garnett theory is normally used in the metal/dielectric nanostructures if 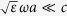 , where *ω* is the frequency of the incident light, *a* is the radius of the metal nanospheres, and *ε* is the permittivity of the medium. Based on this theory, the effective permittivity *ε*_eff_ is generally considered to be an average of the permittivity of the substrate *ε*_*sub*_ and that of the external surrounding medium, *ε*_*ext*_, i.e., 
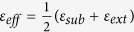
19,20. However, our results indicate that the dipole resonance wavelength of Ag NPs blueshifts or redshifts with increasing thickness of SiO_2_ beneath or overcoating the Ag NPs, respectively. Thus, the effective permittivity cannot be simplified as the average of the different surrounding materials in every case.

Here, Bruggeman effective medium theory is considered to obtain the uniform effective permittivity of the mixed surrounding media^21^,^22^. In three-dimensional Bruggeman’s symmetric effective-medium approximation model, the effective permittivity *ε*_*eff*_ of a two-phase mixture with permittivity and fractions of the components can be expressed as follows:


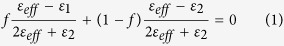


Here *f* is the volume fraction of the first phase with permittivity *ε*_1_ and (1-*f*) is the volume fraction of the second phase with permittivity *ε*_2_. According to Equation (1), the effective permittivity *ε*_*eff*_ is deduced as:


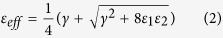


where


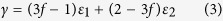


Based on Equations (2) and (3), the uniform effective permittivity of the mixed surrounding media can be calculated with the refractive index n of *n*_*air*_ *=* 1.00, *n*_*silica*_ = 1.46 and *n*_*sapphire*_ = 1.77, the uniform effective permittivity of the surroundings are 1.98, 1.84, 1.75 and 1.66 for the thickness of SiO_2_ beneath the Ag NPs of 5 nm, 10 nm, 15 nm and 20 nm, respectively. While, for the sandwiched Ag NPs, the effective permittivity of the surroundings is 1.93, 2.04, 2.15 and 2.28 for the SiO_2_ beneath the Ag NPs is 10 nm and the SiO_2_ ovecoating Ag NPs of 5 nm, 10 nm, 15 nm and 20 nm, respectively.

Since a surrounding dielectric screens the surface charge, increasing the effective permittivity causes the redshift in the LSPR wavelength. According to the uniform effective permittivity of the mixed surrounding media calculated above, the experimental results can be explained as follows: the silica beneath Ag NPs has a smaller permittivity than the original medium sapphire, and then increasing the thickness of SiO_2_ leading to decrease of the effective permittivity of the surrounding media and thus the blueshift of LSP. While the silica overcoating Ag NPs has a larger permittivity than the original medium air, which gives rise to the increase of the effective permittivity of the surroundings and thus the redshift of LSP.

**Figure 4:**
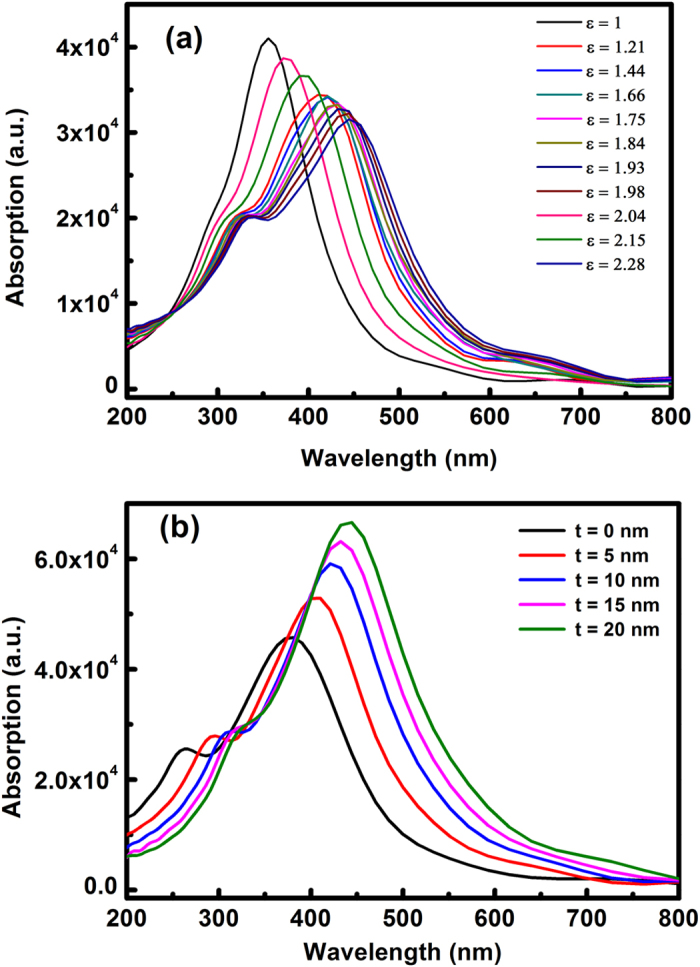
FDTD simulation of the Ag NPs. (**a**) Simulated extinction spectra of Ag NPs surrounded by media with various effective permittivities. (**b**) Simulated extinction spectra of the dimer Ag/SiO_2_ core/shell structure with different SiO_2_ thicknesses.

The time-domain and frequency-domain finite-element method is used to simulate the effects of the cladding dielectric SiO_2_ layer on the resonant wavelengths of the LSPs. The detail descriptions of the numerical calculation are as follows: the semispherical silver model was used corresponding to the experimental results and the dielectric function of silver is referred^23^. In the simulation, uniform effective permittivity was considered because firstly, the permittivity of mixed surrounding media in the experiment can be equivalent to the uniform effective permittivity and secondly, this simulation aims to reveal the physics mechanism behind the shift of the dipole surface plasmon resonance wavelength by changing the permittivity of the surrounding media. Figure 4(a) shows the extinction spectra of Ag NPs embedded in dielectric media with various effective permittivities. Here, an isolated Ag NP with a diameter of 100 nm is considered. The resonant wavelength of the Ag NP redshifts as the effective permittivity increases, which corresponds with our experimental results. In addition, the effect of increasing the SiO_2_ thickness on the interactions of the Ag NPs was also calculated. Figure 4(b) shows the extinction spectra of the Ag NP aggregates with increasing SiO_2_ layer thickness, where a dimer Ag/SiO_2_ core/shell structure is considered, and the Ag NP diameter is 100 nm with a gap distance of 40 nm. Here, the SiO_2_ layer thickness is abbreviated as “t”. The calculated results show that the spectral position of the Ag NP plasmon resonance redshifts and the intensity of the quadrupole resonance of the Ag NPs decreases with increasing SiO_2_ layer thickness, which is also consistent with our experimental results.

In conclusion, the LSPR wavelengths of Ag NPs can be tuned easily and steadily by cladding SiO_2_ dielectric media. The spectral position of the Ag NP plasmon resonance shifts from 470 nm to 410 nm when the SiO_2_ thickness beneath the Ag NPs changes from 5 nm to 20 nm. At the same time, the LSPR wavelength shifts from 450 nm to 490 nm as the SiO_2_ overcoating thickness of the Ag NPs increases from 5 nm to 20 nm. The proportion of SiO_2_ in the surrounding dielectric media is enhanced with increasing SiO_2_ thickness, leading to a change in the effective permittivity and thus a redshift or blueshift in the LSPR wavelength. In addition, the SiO_2_ cladding layer can suppress the asymmetric distribution of the surface charges, and can thus reduce the intensity of the quadrupole plasmon resonance of the Ag NPs. The results presented here provide an easy and stable way to control the resonance wavelengths of LSPs in the blue region for optimum application of surface plasmons in optoelectronic devices.

## Methods

A 6-nm Ag layer was initially deposited on c-plane sapphire by electron beam evaporation at a deposition rate of 0.2 nm/s and a pressure of 5 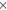 10^−4^ Pa, and was subsequently annealed in N_2_ atmosphere at 300 °C, 500 °C, 600 °C and 800 °C for 5 min. SiO_2_ layers were fabricated by plasma-enhanced chemical vapour deposition (PECVD). For some samples, we grew SiO_2_ with various thicknesses beneath the Ag NPs, while for other samples, SiO_2_ was grown both beneath and on top of the Ag NPs to form a sandwiched Ag structure. The SiO_2_ thickness varies from 0 nm to 20 nm.

The morphologies of the Ag NPs and the SiO_2_ cladding layers were measured by SEM. The optical extinction spectra for all samples were measured using a Shimadzu UV-3101PC scanning spectrophotometer.

## Additional Information

**How to cite this article**: Liu, X. *et al.* Tunable Dipole Surface Plasmon Resonances of Silver Nanoparticles by Cladding Dielectric Layers. *Sci. Rep.* **5**, 12555; doi: 10.1038/srep12555 (2015).
